# Suprascapular Nerve: Is It Important in Cuff Pathology?

**DOI:** 10.1155/2012/516985

**Published:** 2012-11-01

**Authors:** Lewis L. Shi, Michael T. Freehill, Paul Yannopoulos, Jon J. P. Warner

**Affiliations:** ^1^Department of Orthopaedic Surgery and Rehabilitation Medicine, The University of Chicago Hospitals, 5841 South Maryland Avenue, MC 3079, Chicago, IL 60637, USA; ^2^Department of Orthopaedic Surgery, Wake Forest University Baptist Medical Center, Medical Center Boulevard, P.O. Box 1070, Winston-Salem, NC 27157-1070, USA; ^3^Division of Shoulder Surgery, Massachusetts General Hospital, Yawkey Center 3G, 55 Fruit Street, Boston, MA 02114, USA

## Abstract

Suprascapular nerve and rotator cuff function are intimately connected. The incidence of suprascapular neuropathy has been increasing due to improved understanding of the disease entity and detection methods. The nerve dysfunction often results from a traction injury or compression, and a common cause is increased tension on the nerve from retracted rotator cuff tears. Suprascapular neuropathy should be considered as a diagnosis if patients exhibit posterosuperior shoulder pain, atrophy or weakness of supraspinatus and infraspinatus without rotator cuff tear, or massive rotator cuff with retraction. Magnetic resonance imaging and electromyography studies are indicated to evaluate the rotator cuff and function of the nerve. Fluoroscopically guided injections to the suprascapular notch can also be considered as a diagnostic option. Nonoperative treatment of suprascapular neuropathy can be successful, but in the recent decade there is increasing evidence espousing the success of surgical treatment, in particular arthroscopic suprascapular nerve decompression. There is often reliable improvement in shoulder pain, but muscle atrophy recovery is less predictable. More clinical data are needed to determine the role of rotator cuff repair and nerve decompression in the same setting.

## 1. Introduction

The suprascapular nerve provides sensory innervation to the posterosuperior aspect of the shoulder and motor innervation to supraspinatus and infraspinatus muscles. Dysfunction of the suprascapular nerve is intimately associated with rotator cuff pathology; nerve dysfunction can lead to cuff disease and vice versa. Suprascapular neuropathy is typically due to compression or traction of the nerve, and this can result in a spectrum of clinical symptoms, including pain in the posterosuperior aspects of the shoulder and weakness in forward flexion and external rotation. The various presentations of suprascapular neuropathy can make its diagnosis a challenge. Historically, suprascapular neuropathy has been viewed as a diagnosis of exclusion, but with recent advances, there is better understanding of both the etiology and treatment options, particularly relating to rotator cuff pathology.

## 2. Etiology of Suprascapular Neuropathy

**Figure 1:**
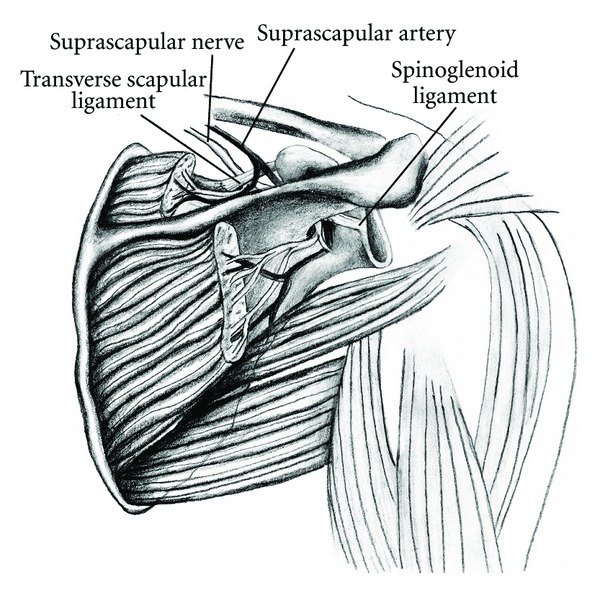
Illustration of suprascapular nerve anatomy. The nerve courses under the transverse scapular ligament and the spinoglenoid ligament with motor branches to the supraspinatus and infraspinatus. From Boykin et al., [50] permission granted.

The suprascapular nerve arises from the upper trunk of the brachial plexus. It travels posterior to the clavicle and enters the suprascapular notch by passing beneath the transverse scapular ligament. The motor branches innervate the supraspinatus, and the nerve continues past the spinoglenoid notch and innervates the infraspinatus (Figure 1). 

**Figure 2:**
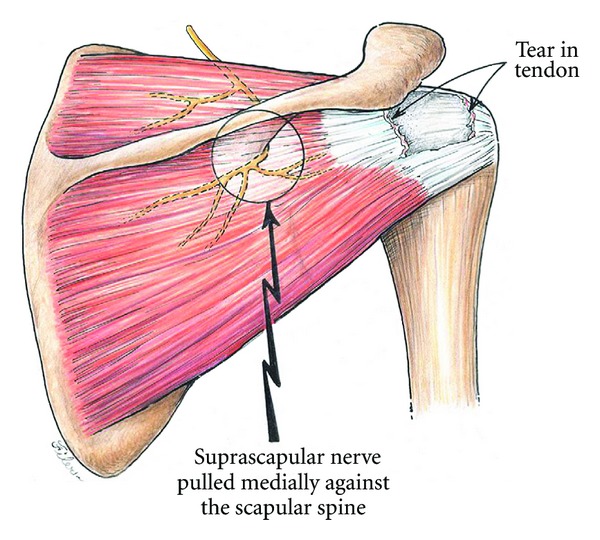
Medial traction on the suprascapular nerve secondary to a massive rotator cuff tear involving the supraspinatus and infraspinatus. From Costouros et al. [7]. Permission granted.

Suprascapular neuropathy can result from traction injury or compression of the nerve at various points of its path. The most common cause of the neuropathy is due to traction injury on the nerve produced by a retracted superior or posterior rotator cuff tear. As supraspinatus and/or infraspinatus retract, there is increasing tension on the suprascapular nerve at the suprascapular notch or spinoglenoid notch (Figure 2). Albritton et al. demonstrated in a cadaveric study that increasing retraction of the supraspinatus tendon led to a reduction in the angle between the suprascapular nerve and its first motor branch and thus increased tension [1]. Electrodiagnostic findings of suprascapular neuropathy have also been reported in eight patients with a massive rotator cuff tear [2]; however, Vad et al. found only an 8% rate of suprascapular neuropathy (2/25) in patients with a full-thickness rotator cuff tear and muscle atrophy [3].

Various studies have shown that the tension in the rotator cuff muscle and tendon plays an important role in the health of the suprascapular nerve. Greiner et al. reported in cadavers that the maximum lateral advancement of a retracted rotator cuff tear is between 1 and 3 cm; with increased advancement, the neurovascular pedicle is placed under heightened tension [4]. Warner et al. also demonstrated that tension on the motor branches of the suprascapular nerve increases with rotator cuff advancement of greater than 3 cm [5], while another study demonstrated tension on the medial motor branches of this nerve with only 1 cm of advancement [4]. Hoellrich et al. did not find any electrodiagnostic suggestions of suprascapular neuropathy after repairs of massive rotator cuff tears in nine patients with an average advancement of 2.5 cm (range 2.0 to 3.5 cm) and concluded that the tendons can be mobilized and advanced up to 3.5 cm without risk to the nerve [6].

These are published reports that suggest suprascapular neuropathy associated with retracted rotator cuff tear may partially or completely resolve with repair of the rotator cuff. Mallon et al. reported two of four patients with suprascapular neuropathy and a massive retracted rotator cuff tear showed reinnervation potentials after partial arthroscopic rotator cuff repair [2]. Costouros et al. reported that all 6 patients with preoperative electrodiagnostically confirmed suprascapular neuropathy showed nerve recovery after partial or complete rotator cuff repair [7]. 

Besides retracted rotator cuff tears, there can be other causes of suprascapular neuropathy. Repetitive overhead athletes have been reported to experience neuropathy secondary to traction and microtrauma [8–10]. The mechanism is tightening of the spinoglenoid ligament when the shoulder is in a position of overhead throwing [11]. Ossification of the transverse scapular ligament or spinoglenoid ligament may increase the risk of suprascapular neuropathy [10, 12–15]. Compression of the nerve can also occur at either the suprascapular or spinoglenoid notch by soft-tissue or bone tumor, or a cyst secondary to a labral or capsular injury [16–18]. Other less frequent causes of suprascapular neuropathy include brachial neuritis [19], after glenohumeral dislocation [20–22], after fractures about the shoulder girdle [23, 24], and penetrating or iatrogenic injury to the nerve [25].

## 3. Prevalence and Incidence of Suprascapular Neuropathy

Zehetgruber et al. published a meta-analysis reporting only 88 reports of this condition over a 42-year period [26]. Other reports in the literature suggest suprascapular neuropathy accounts for 1% to 2% of all shoulder pain [27]. Boykin et al. reported a 4% (40/937) incidence of suprascapular neuropathy in a tertiary shoulder referral practice [28]. Literature from the past decade has provided a prevalence ranging from 12% to 33% in athletic populations and 8–100% in patients with massive rotator cuff tears [2, 3, 7, 8, 12].

## 4. Diagnosis

Patients typically describe an insidious onset of dull, aching pain localized to posterosuperior aspect of the shoulder. Weakness and fatigue with overhead activities are common complaints. The physical finding of atrophy of the supraspinatus and/or infraspinatus fossa can be visualized on inspection. In long-standing cases, the teres minor muscle may compensate for the loss of the infraspinatus muscle and maintain nearly normal strength in external rotation [8]. The strength of supraspinatus and infraspinatus should be separately graded, preferably quantitatively with a hand-held dynamometer. Targeted examination of the labrum and cervical spine should also be carried out if suprascapular neuropathy is part of the differential diagnosis.

**Figure 3:**
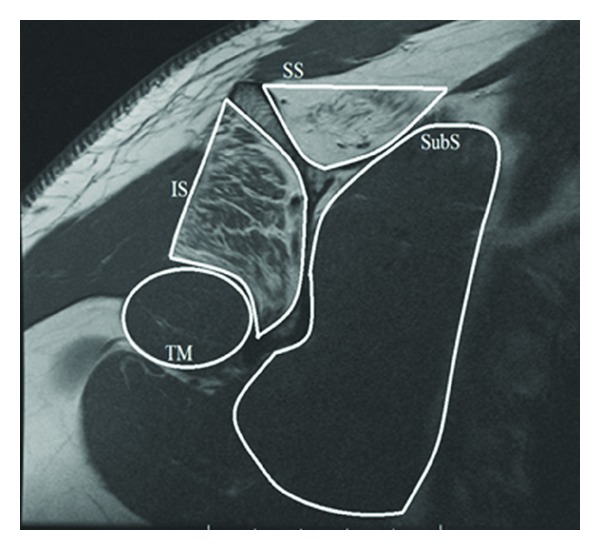
T1 sagittal oblique MRI image of rotator cuff muscles, demonstrating fatty infiltration of the supraspinatus and infraspinatus muscles. IS, infraspinatus; SB, subscapularis; SS, supraspinatus; TM, teres minor.

**Figure 4:**
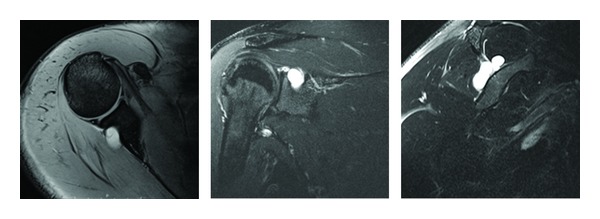
Axial, coronal, and sagittal MRI images of a patient with multiple paralabral cysts in the suprascapular notch and spinoglenoid notch.

Radiographs are recommended to assess for fracture, callus formation around the nerve, bone tumor, and osseous dysplasia. Two additional dedicated views include the Stryker notch view which can show the suprascapular notch and the “suprascapular notch view,” with the beam directed 15 to 30 degrees cephalad, that allows for the evaluation of osseous variants. Magnetic resonance imaging (MRI) can evaluate the rotator cuff muscles and tendons, including fatty infiltration and atrophy [29] (Figure 3). It can also detect any space-occupying lesions, such as paralabral cysts [30, 31] (Figure 4). 

The gold standard for diagnosis and confirmation of suprascapular nerve injury is electromyography (EMG) and nerve conduction velocity (NCV) studies. Indications for these studies should include (1) persistent posterosuperior shoulder pain without a diagnosis; (2) atrophy and/or weakness of the supraspinatus and/or infraspinatus with no evidence of a rotator cuff tear; (3) MRI demonstrating muscle edema suggestive of nerve injury; (4) massive rotator cuff tendons with retraction and traction on the nerve.

Electrodiagnostic studies have normative values which have been established and published, but there is variability, and criteria for interpretation can vary between centers and practices [32, 33]. Typically EMG changes consistent with denervation are fibrillations and sharp waves and slower NCV and increased latency from Erb's point to supraspinatus and infraspinatus. Nerve conduction velocities and electromyography studies have been reported as 91% accurate (72/79) in detecting nerve injury associated with muscle weakness [34].

Finally, a fluoroscopically guided injection of local anesthetic into the region of the suprascapular nerve may be useful to evaluate for pain relief in patients for whom the findings of these diagnostic studies are negative or equivocal and continue to have unexplained symptoms.

## 5. Nonoperative Treatment 

If suprascapular neuropathy is diagnosed secondary to a labral tear with paralabral cyst or rotator cuff tear, the concomitant pathology may dictate the treatment. In the case of an isolated suprascapular neuropathy, nonoperative modalities can be attempted initially, including activity modification, nonsteroidal anti-inflammatory drugs, and physical therapy [35, 36]. Black and Lombardo reported 4 cases of patients with suprascapular neuropathy affecting the infraspinatus. Patients improved with nonoperative treatment over 6 months to 1 year [37]. Another report noted 4 patients with isolated suprascapular neuropathy improving with therapy alone and recommended 6 to 8 months of nonoperative treatment [38]. Martin et al. reported on 15 patients with isolated suprascapular neuropathy managed nonoperatively with average followup of almost 4 years. Five patients had an excellent result, seven had a good result, and 3 required surgical intervention [35]. 

## 6. Operative Treatment 

If an isolated suprascapular neuropathy (without concomitant pathology) has failed a course of conservative management, surgical intervention with suprascapular nerve decompression is offered [39–44]. If the neuropathy is diagnosed in combination with rotator cuff tears or labrum tears with paralabral cysts, surgical intervention is often preferred upon diagnosis. There is debate as to the necessity of transverse scapular ligament release if concomitant pathology is addressed, with some authors recommending the release [39, 45], and other authors reporting resolution of suprascapular neuropathy without nerve decompression after isolated rotator cuff or labral repair [7, 46, 47].

Release of the transverse scapular ligament can be performed via either an open or arthroscopic technique. Generally, with open technique, few complications have been reported with most patients experiencing resolution of pain and improvement of muscle strength. However, reversal of muscle atrophy is not always observed. Kim et al. reported 90% of patients with profound muscle weakness showing improvement in supraspinatus muscle strength to grade 4 or better; however, improvement in infraspinatus muscle strength was less predictable. Additionally, eighty-eight percent of patients with severe pain reported improvement [43]. Another study report, an 89% rate of good-to-excellent results after open suprascapular nerve decompression; again, resolution of muscle atrophy was less predictable at only 52% [42].

The technique of arthroscopic suprascapular nerve decompression has been described elsewhere [39, 48, 49]. Lafosse et al. reported their early results of arthroscopic suprascapular nerve decompression at the suprascapular notch in 10 patients [39]. At 6 months after surgery, nine out of 10 patients graded their outcome as excellent with complete relief of pain, seven of 10 had complete normalization of EMG/NCV, and 2 others showed partial recovery of the nerve. Shah et al. reported on 27 patients who underwent arthroscopic suprascapular nerve decompression at the suprascapular and/or spinoglenoid notch [40]. Seventeen out of 24 (71%) experienced significant pain relief at 9 weeks after surgery; their subjective shoulder value and American Shoulder and Elbow Society shoulder scores significantly improved. 

## 7. Conclusion

With growing body of published literature on suprascapular nerve and rotator cuff, there is better understanding of the relationship of the function and dysfunction of each. The incidence of suprascapular neuropathy may be on the rise due to improved recognition and diagnosis of this entity. In patients who exhibit vague posterosuperior shoulder pain, unexplained atrophy, and weakness of supraspinatus and infraspinatus, and in all patients with retracted rotator cuff tears, suprascapular neuropathy should be part of the differential diagnoses. MRI and EMG are indicated to evaluate the rotator cuff and the health of the nerve; fluoroscopically guided injections to the suprascapular notch can also be considered as a diagnostic option. Both open and arthroscopic suprascapular nerve decompressions have yielded good results. Additional high level studies are needed to examine the role of concomitant rotator cuff repair and nerve decompression in shoulder surgery.
